# Comprehensive Validation of Snapback Primer-Based Melting Curve Analysis to Detect Nucleotide Variation in the Codon 12 and 13 of KRAS Gene

**DOI:** 10.1155/2018/8727941

**Published:** 2018-10-10

**Authors:** Qunfeng Zhang, Yiqiao Du, Xinju Zhang, Zhihua Kang, Ming Guan, Zhiyuan Wu

**Affiliations:** ^1^Department of Clinical Laboratory, Huashan Hospital, Fudan University, 200040, China; ^2^Baoshan Hospital of Integrated Traditional Chinese and Western Medicine, Shanghai, 201999, China; ^3^Central Laboratory, Huashan Hospital, Fudan University, Shanghai 200040, China; ^4^Department of Clinical Laboratory, Huashan Hospital North, Fudan University, Shanghai, 201907, China

## Abstract

**Background:**

KRAS genotyping in tumor samples is a decisive clinical test for the anti-EGFR therapy management. However, the complexity of KRAS mutation landscape across different cancer types and the mosaic effect caused by cancer cellularity and heterogeneity make the choice of KRAS genotyping method a challenging topic in the clinical practice.

**Methods:**

We depicted the landscape of somatic KRAS mutation in 7,844 primary tumors and 10,336 metastatic tumors across over 30 types of cancer using the Cancer Genome Atlas (TCGA) and Integrated Mutation Profiling of Actionable Cancer Targets (MSKCC-IMPACT) databases, respectively. A snapback primer assay based on melting curve analysis was developed to detect the most common somatic mutations in KRAS codons 12 and 13. The sensitivity and accuracy of the method was validated by genotyping 100 colorectal cancer (CRC) samples, in comparison with Sanger sequencing and T-A cloning sequencing.

**Results:**

Pancreas adenocarcinoma (somatic mutation frequency 90.6%), colorectal adenocarcinoma (42.5%), and lung adenocarcinoma (32.6%) are the top three most KRAS mutant primary cancer types. The metastatic tumors showed a higher prevalence (90.99% versus 66.31%) and diversity of KRAS mutation compared with the primary tumors. Mutations in codons 12 and 13 are the predominant genetic alteration in KRAS (84.15% for TCGA and 86.13% for MSK-IMPACT). Moreover, KRAS mutation is highly correlated with the overall survival of patients with metastatic cancer. The snapback primer assay showed a more favorable performance in enriching and detecting the KRAS codon 12 and 13 mutation (1% mutation load) compared with Sanger sequencing (20% mutation load and 7% false-negative rate).

**Conclusions:**

KRAS mutation pattern is highly diverse among different cancer types and is associated with the survival of patients with metastatic cancers. The snapback primer assay is a reliable, sensitive method to detect the major mutant KRAS alleles, which might facilitate the effective cancer treatment decisions.

## 1. Introduction

KRAS, as a member of the RAS family, is a small GTPase that frequently mutated in a wide range of cancers including pancreatic [1], colorectal [2], and lung cancers [3]. Physiologically, KRAS catalyzes the hydrolysis of GTP to GDP and cycles between an active (GTP-bound) and an inactive (GDP-bound) state. Once activated, KRAS promotes the regulation of cellular proliferation through the receptor tyrosine kinase- (RTK-) MAPK/ PI3K signaling cascades [4].

Transforming mutations in KRAS are frequently found in three major hotspots, G12, G13, and Q61. The codons 12 and 13 mutations occur in the GTPase domain, and the codon 61 mutations debilitate the NF1 binding ability of KRAS. All these mutations cause an RTK independent constitutive activation of the protein and intracellular signaling including the RAF/MEK/ERK and PI3K/AKT/mTOR pathways [5–7].

Besides its crucial role in the tumor development, the KRAS mutational status is also critical for the antiepidermal growth factor receptor (EGFR) therapy management in colorectal cancers, which has greatly improved the clinical outcome of the disease in the past decade [8–10]. It has been widely reported that the KRAS mutation can cause the failure of anti-EGFR and chemo drugs combined therapy since the predominant gain-of-functionally mutant variants can bypass the EGFR depletion [11–13]. Moreover, the KRAS mutations have been shown in lung cancer to be a negative predictor of EGFR inhibitors [14, 15]. Given this evidence, the KRAS mutation testing is approved by food and drug administration (FDA) for treatment of KRAS mutation-negative (wild-type), EGFR-expressing metastatic colorectal cancer, and is required by European medicines agency (EMA) before the initiation of anti-EGFR therapy.

There are currently several technically available modes for the KRAS mutation detecting practice, such as the conventional Sanger sequencing [16], TaqMan real-time PCR [17], and high resolution melting analysis (HRMA) [18]. Besides, molecular technologies have been developed to increase the sensitivity of mutant allele identification, such as amplification refractory mutation system (ARMS)[19], peptide nucleic acid (PNA) clamp PCR assay [20], coamplification-at-lower denaturation-temperature (COLD) PCR[21]], which enrich the mutant allele during upstream oligonucleotide amplification, and the ones using ultra resolution discriminating technologies as pyrosequencing[22], next-generation sequencing (NGS) [23], and droplet digital PCR (ddPCR)[24].

However, there still remain two major challenges in the precision medicine implementation of KRAS. First, the comprehensive study of KRAS mutation landscape and the clinical relevance of the mutations across the different major cancer types is still limited, although the mutation hotspot analysis spanning the whole KRAS gene has been reported in different cancer types separately, especially in PAAD, COAD, and LUAD. On the other hand, the universal method for KRAS mutation detecting needs to be determined due to the high diversity of KRAS mutation and tumor cellularity in cancers [25], especially in PAAD [26]. Hence, a thorough survey of KRAS somatic mutation and the evaluation of a fast, sensitive, economical, and reliable clinical assay to detect these mutations has become increasingly important.

In this study, we assessed the mutational landscape and somatic mutation burden of the KRAS gene across 32 major primary cancer types and metastatic cancers with 33 different tissue origins using DNA-SEQ data from The Cancer Genome Atlas (TCGA) and Memorial Sloan Kettering Cancer Center Integrated Mutation Profiling of Actionable Cancer Targets (MSKCC-IMPACT) database. Meanwhile, survival analysis stratified by the KRAS mutation status was performed to validate the prognostic value of KRAS mutation detecting. A snapback primer based high resolution melting analysis (HRMA) system was developed to detect the most prevalent KRAS mutations in codons 12 and 13 after evaluation of the practicability of currently available mutation discriminating technologies.

## 2. Materials and Methods

### 2.1. Database and Data Extraction

The copy number alteration, somatic DNA mutation status, mutant allele burden, and survival data of TCGA and MSKCC-IMPACT database were obtained and extracted from the cBioPortal website (http://www.cbioportal.org/index.do). The interval between the date of initial surgical removal to the date of patient death or last contact was calculated as overall survival duration. The interval between the date of initial surgical removal and the date of tumor progression/recurrence was calculated as disease-free survival.

### 2.2. Statistic Analysis and Survival Analysis

The genomics and clinical data were analyzed with standard statistical tests, including the K-S test and log-rank survival test. Statistical significance was defined as P<0.05. The survival analyses were performed using cBioPortal interface.

### 2.3. Patient Samples and DNA Preparation

Resected primary colorectal cancers (CRCs) were obtained from 100 patients in Huashan Hospital of Fudan University, and these tissues were stored in liquid nitrogen before DNA extraction. Written informed consent was received from all the participants. DNA was extracted from the tissue specimens with the DNA QIAamp DNA Extraction Kit (Qiagen, Hilden, Germany) and diluted with deionized distilled water (ddH_2_O) to a final concentration of 15-25 ng/*μ*L. In compliance with Helsinki Declaration of 1975 as revised in 1996, this study was approved by the Institutional Review Board of Huashan Hospital.

### 2.4. Construction of Artificial Plasmids

KRAS wild-type and mutant plasmids (G12D, G12V, G12S, G12C, G12A, and G13D) were constructed with pMD19-T simple vector using the QuikChange® Lightning Site-Directed Mutagenesis Kit (Agilent Technologies, Lexington, MA), respectively, according to the manufacturers' instruction. Meanwhile, to validate the discriminating power of snapback primer systems, the plasmid fragments with 2 and 3 nucleotide variations (GGT>GCA, GTG>CAC) were also synthesized by Shanghai Xiangyin Biotechnology Co. Ltd. All these artificial plasmids were verified by Sanger sequencing and were finally diluted to 10^6^ copies per microliter.

### 2.5. Snapback Primer Design and HRM Analysis

The snapback primer system was developed on a 9700 GeneAmp PCR system (Applied Biosystems) and was designed to amplify a flanking region of 118 bp of KRAS gene flanking codon 12 and 13. The primer sequences are as follows: Snapback primer (5′-at*CCTACGCCACCAGCTCCA*AGGCCTGCTGAAAATGACTGAA-3′) and limiting primer (5′-TGTTGGATCATATTCGTCCACAAAATG-3′). For the snapback primer, the bases in uppercase italic signify the probe element and the nucleotides in uppercase at the 3′ end stand for the conventional annealing primer. Thus, during PCR annealing, the 3′ primer will anneal to the template for the subsequent extension while the 5′snapback probe will combine with the target sequence of PCR amplicon, which will finally form the stem-loop secondary structure. The 5′-terminal of the snapback primers was blocked with 2 nucleotides (bases in lowercase) that mismatched the target sequence so as to eliminate the unfavorable extension of the snapback probe during PCR. The limiting primer was designed based on the principle of linear-after-the-exponential PCR (LATE-PCR)[27], making the limiting primer either longer or higher in the percentage of guanine and cytosine (G+C) relative to the excess primer. As a result, both the excessive snapback primer and the limiting primer can ensure high efficiency and specificity to produce single strand amplicon during the asymmetric PCR procedure.

Asymmetric PCR was performed in a 20 *μ*L reaction volumes containing 1U TaKaRa Ex Taq HS (TaKaRa BIO, Shiga, Japan), 2 *μ*L 10×Ex Taq buffer (Mg^2+^free), 2.5 mM MgCl_2_, 2.5 mM deoxynucleotide triphosphate (dNTP) Mixture, 0.5 *μ*M of the snapback primer, 0.05 *μ*M of the limiting primer, 1.5 *μ*M SYTO-9 DNA dye (Invitrorgen, Carlsbad, CA), and 10^6^ copies of plasmid. The PCR was performed on a 9700 GeneAmp PCR system for 70 cycles with an initial denaturation at 95°C (10 mins hold), denaturation at 95°C (0 s hold), annealing at 56°C (0 s hold), and a 2°C/s ramp to the extension temperature of 68°C (0 s hold). The HRM analysis was performed on the RotorGene Q (Qiagen). The products were heated for denaturation at 95°C for 2 mins, followed by cooling down to 40°C for 2min to facilitate the hybridization of snapback probe, and then melted at a ramping rate of 0.5°C /sec from 50°C to 92°C. HRM curve analysis was performed with the Rotor-Gene Q 1.7 software.

### 2.6. Validation of Mutant Allele Enrichment during Snapback Primer PCR

So as to validate the mutation enrichment during snapback primer-guided nucleotide amplification, serial dilutions of KRAS G12A mutant plasmid containing 100%, 10%, 1%, and 0% mutant allele were amplified with both the snap-back PCR primer set and the normal PCR primers (forward primer: 5′-AGGCCTGCTGAAAATGACTGAA-3′; reverse primer: 5′-TGTTGGATCATATTCGTCCACAAAATG-3′) using the thermocycling condition described in the Section 2.5, respectively. The PCR products were further subjected to Sanger sequencing. The abundance of the mutant allele in the products was assessed by the relative fluorescent signal intensity produced by the mutant nucleotide.

### 2.7. Analytical Sensitivity of Snapback Primer System

Representative KRAS mutant plasmid (G12D) was mixed in various ratios with the KRAS wild-type plasmid to obtain the standards of 100%, 50%, 10%, 1%, 0.1%, and wild-type control. Then the analytical sensitivity of the assay was evaluated by detecting these serial standards and compared with Sanger sequencing PCR.

### 2.8. DNA Sequencing and T-A Cloning Assays

All the DNA of CRC tissues was subjected to bidirectional Sanger sequencing, the sequences of the primers as follows: forward (5′-ACAGTTCATTACGATACACG-3′) and reverse (5′-CCCAAGGTACATTTCAGATAAC-3′). All the mutation-negative amplicons identified by direct sequencing were subjected to T-A cloning. The 450bp amplicon was separated with 1.5% agarose electrophoresis and purified through the Qiaquick gel purification kit (Qiagen) before being combined with pMD19-T simple vector master mix (Takara BIO). Then the vector plasmids with cloned insert were transformed into DH5*α* competent E. coli cells and multiplied in the Luria-Bertani (LB) broth and spread onto the IPTG/x-GAL (Invitrogen) coated ampicillin-LB agar dishes according to the instructions of the manufacturer. After 37°C incubation for 16hours, the white clones were picked up and again enriched in the ampicillin-LB broth at 37°C overnight. The monoclonal colonies collected were extracted with Plasmid Mini Kit (Qiagen) according to the manufacturer's instruction. All the Sanger sequencing assays were performed on an Applied Biosystems PRISM 3130 genetic analyzer in the Invitrogen Laboratory of Technical Services (Shanghai, China).

### 2.9. Validation of Intra- and Interassay Precision of Snapback Primer System

Plasmids with 1% burden of KRAS G12D or G13D mutation was used as standard for the precision validation experiment. The precision was determined as the coefficient of variation (CV) of the mutant allele melting temperature calculated by the Rotor-Gene Q V1.7 software (Qiagen). The intra-assay precision validation was performed by parallelly detecting twenty 1% mutant allele standard, 100%, and 0% mutant allele plasmid in one batch of experiment. The interassay precision validation was carried out as a 5-day continuous experiment detecting four 1% mutant allele standard, 100%, and 0% mutant allele control.

## 3. Results

### 3.1. Landscape of KRAS Somatic Mutations in Cancer

We extracted the genetic alterations of KRAS gene, including somatic mutation and copy number alteration (CNA) using the DNA-SEQ data from TCGA and MSKCC-IMPACT database. In the TCGA cohort, the genetic alteration information was collected from 7,844 primary tumor tissue samples across 32 cancer types. Meanwhile, in the MSK-IMPACT cohort, the genetic alteration information was obtained from 10,336 metastatic tumor samples across 33 cancer types.

**Figure 1:**
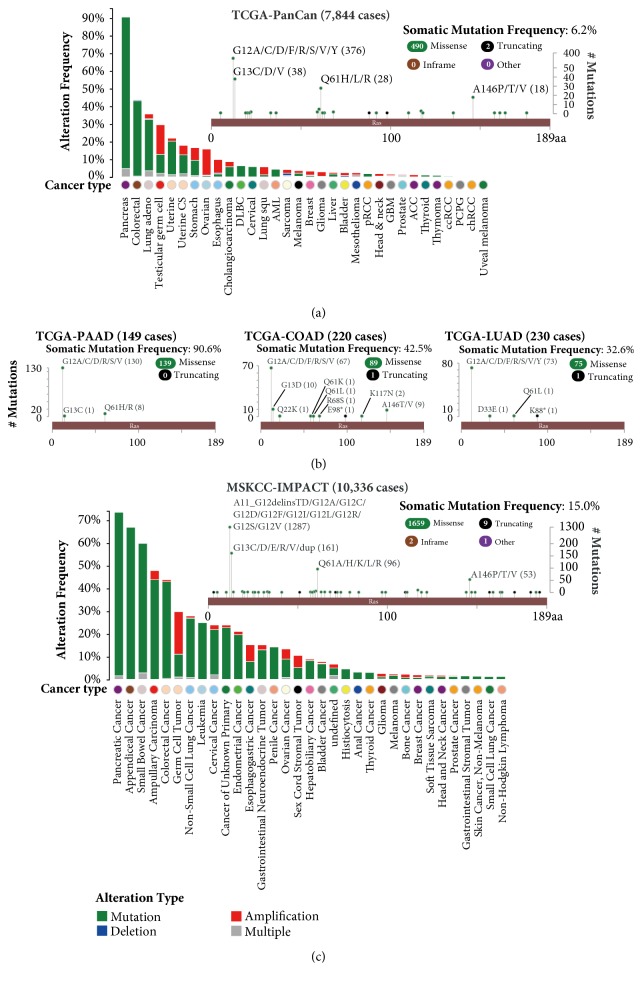
Mutation Landscape of KRAS in TCGA (7,844 primary tumors across 32 cancer types) and MSK-IMPACT (10,366 metastatic cancers) cohort. (a) Onco-print of KRAS genetic alterations across 32 types of primary tumors in TCGA. Somatic mutations are the dominating type of genetic alteration. PAAD, COAD, and LUAD are the top 3 KRAS most mutant cancer types. Copy number amplification are frequently found in TGCT and OV. (b) Lollipop plot of the KRAS mutational hotspot in the TCGA PAAD, COAD, and LUAD cohort. The KRAS mutation is highly prevalent in PAAD. Codons 12 and 13 are the most prevailing mutation hotspot. COAD showed a relatively high KRAS mutation pattern diversity. (c) Onco-print of KRAS genetic alterations in the MSKCC-IMPACT cohort. Somatic mutations are the most common type of genetic alteration across metastatic cancers originated from 33 types of tissue primaries. Both the frequency and the diversity of the mutations are higher than those in the TCGA cohort.

In the TCGA primary tumor samples, pancreas adenocarcinoma (PAAD) is the most KRAS mutant cancer type (somatic mutation frequency 90.6% of 149 cases), followed by colorectal adenocarcinoma (COAD, 42.5% of 220 cases) and lung adenocarcinoma (LUAD, 32.6% of 230 cases) (Figure 1(a)). COADs harbor the most diverse mutation pattern, with 89 missense mutations and 1 truncating mutation found in amino acids of G12, G13, Q22, Q61, R68, E98, K117, and A146. The mutations in PAAD are confined to 3 mutation hotspots (G12, G13, and Q61), although PAADs have the highest prevalence of KRAS mutation (Figure 1(b)). Besides the mutations, copy number amplification the gene can also be observed in around 20% of the testicular germ cell tumors (TGCTs) and ovarian cancers (OVs), which may also confer the tumor cells to a KRAS gain-of-function shift in the cellular phenotype.

Compared with the primary tumors in the TCGA cohort, the metastatic tumor tissues in the MSK-IMPACT cohort showed a higher genetic alteration prevalence (K-S test, p<0.05). Moreover, the metastatic tumors are also more somatic mutation-driven other than CNA driven, as the percentage of mutation out of total genetic alteration in metastatic tumors (90.99%) is higher than those in the primary tumors (66.31%). The mutation pattern is also more complicated in the IMPACT cohort. Besides from the high prevalence of missense/truncating mutation, two inframe mutations (G13dup and Q61_S65dup) were found in metastatic tumor tissues originated from mesonephric carcinoma and PAAD respectively, and the KRAS-SOX5 gene fusion was also identified in a metastatic tumor with unknown primary (Figure 1(c)).

In accordance to the previous reports [1–3, 5], our survey confirmed that the somatic mutations in codons 12 and 13 are the predominant genetic alteration in KRAS with regard to the prevalence (84.15% for the TCGA and 86.13% for MSK-IMPACT). However, these mutations are still genetically diversified, as 11 types of codons 12 and 13 mutations were found in the primary tumors, while 16 types of codons 12 and 13 mutation were found in the metastatic tumors. Besides the diversity of the hotspot mutations, we further found 4 TCGA PAAD and 7 MSK-IMPACT tumors that have simultaneous mutations in codons 12 and 13 (Table 1), underlying the cellular and molecular heterogeneity of tumor tissues.

### 3.2. KRAS Mutation Is Associated with the Poor Survival of Patients with Metastatic Tumors

**Figure 2:**
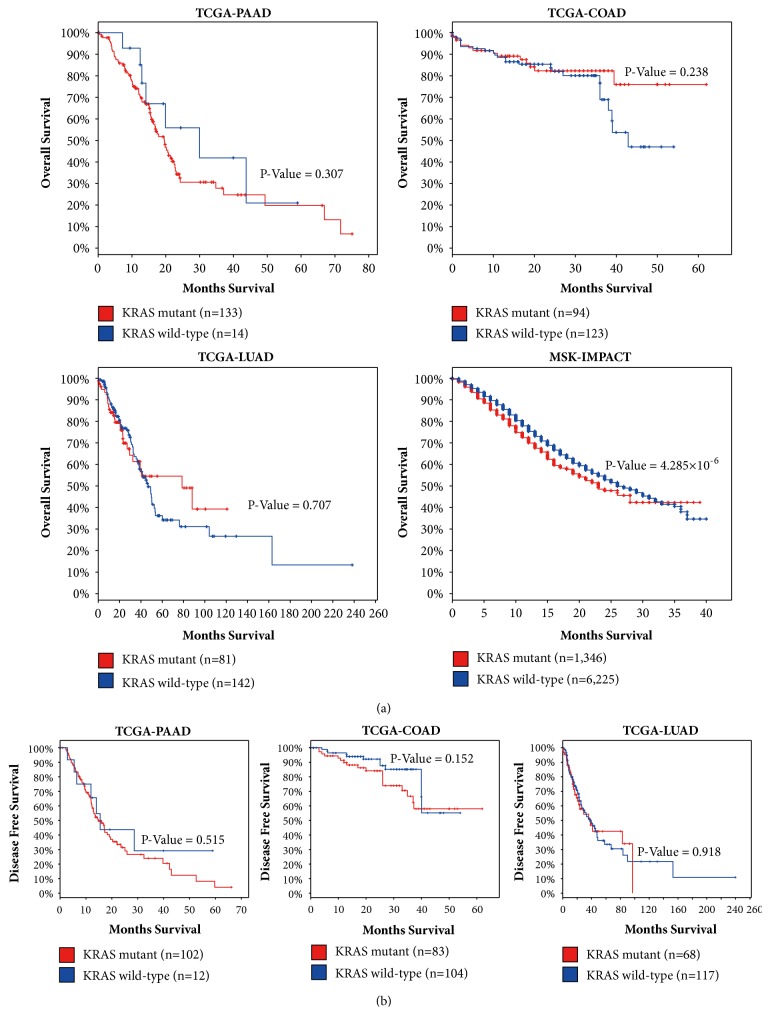
KRAS mutations are associated with poor survival of patients with metastatic tumors other than patients with primary tumors. Log-rank survival curves for overall survival (a) and disease-free survival (b) durations of KRAS mutant (red) and KRAS wild-type (blue) patient in TCGA-PAAD, TCGA-COAD, TCGA-LUAD, and MSKCC-IMPACT cohorts. Metastatic cancer with KRAS mutation showed significantly reduced overall survival compared with their KRAS wild-type counterparts.

Given the comprehensive genetic background of KRAS alteration across different cancer types, we further tested whether KRAS gene mutation will influence the prognosis of the disease. We extracted the overall survival (OS) and disease-free survival (DFS) data from the TCGA PAAD, LUAD, and COAD cohort and the OS data from the MSK-IMPACT database. Log-rank survival analysis was then performed after stratifying the patients with KRAS mutation status. Although there is no difference between the survival of KRAS mutant primary tumors and KRAS wild-type primary tumors, KRAS mutant metastatic tumor showed poorer survival versus their KRAS wild-type counterparts (P=4.285 × 10^−6^) (Figure 2), further suggesting the importance of KRAS mutation detection in the clinical management of metastatic tumors.

### 3.3. Detection of KRAS Codons 12 and 13 Mutations Using Snapback Primer High Resolution Melting Analysis

Given the fact that, over 80% of the KRAS mutations in both primary and metastatic tumors were observed as missense mutation in codons 12 and 13 and these mutations involved 17 mutation types (Figure 1), we decided to develop a mutation screening technique based on snapback primer high resolution melting analysis, which can potentially identify all the gene alterations in the codons 12 and 13 of KRAS gene.

**Figure 3:**
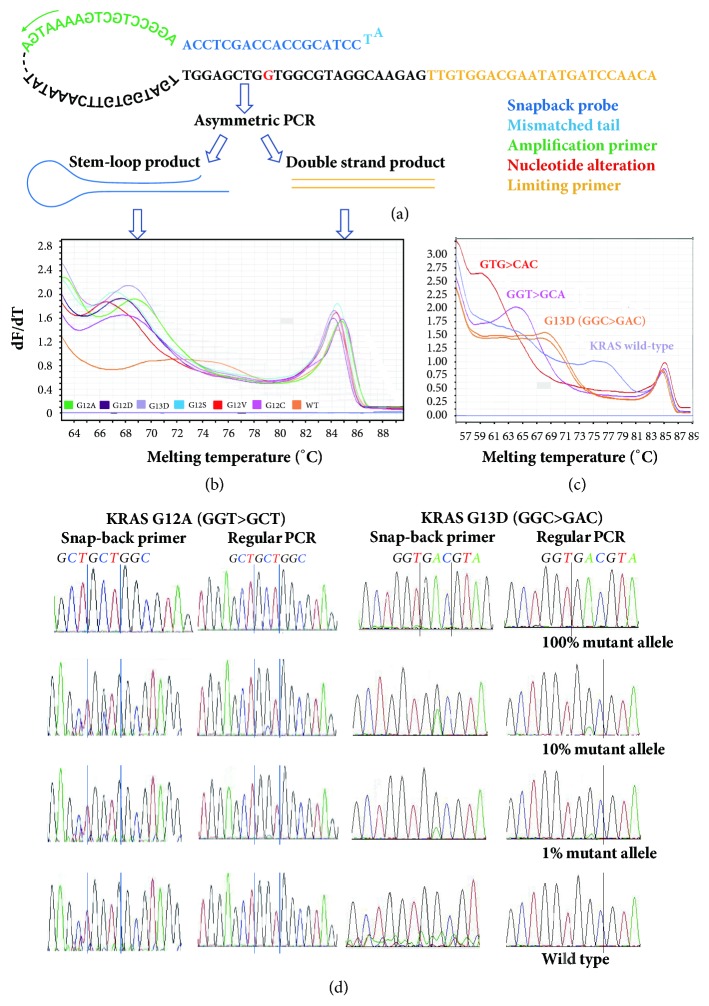
Establishment of a snapback primer PCR based HRMA detecting KRAS codons 12 and 13 mutations. (a) Design of snapback primer HRMA for KRAS genotyping. A probe's element with 18 nucleotides was designed totally complementary to the target sequence. In addition, a 2-bp mismatch at the 5′end of snapback primer was intentionally used to block further extension of the snapback hairpin. When asymmetric PCR conditions were optimized, the stem-loop hairpins and double-strand DNA amplicons were formed. The melting transitions were processed by plotting the negative derivative of fluorescence versus the melting temperature. At the moment, snapback melting peaks signified targeted genotyping. (b) Snapback probe assay for the detection of KRAS codons 12 and 13 mutations. The negative derivative (dF/dT) plot of melting curve consists of two melting regions. Stem-loop hairpin melting peaks for mutation identification were located in the region between 66°C to 70°C, and the double-strand amplicon as DNA template amplification control was situated at ( 84±0.5°C). (c) Discriminating power of the snapback primer assay. Melting curve profile of three plasmids (G13D, GGA>GCA, and GTG>CAC) revealed the specificity of the snapback primer system for detecting single base variation (66°C-70°C), with the at least 2-degree difference in the melting peaks. (d) Enrichment of snapback primer amplification. 1% dilution of the KRAS G12A or KRAS G13D mutant allele can be detected by Sanger sequencing, while the same sequencing signal of a mutant allele can only be observed in the PCR products with 10% mutant allele load.

A schematic of genotyping by snapback primer-based HRM is showed in Figure 3(a). The snapback primer was composed of two parts, the snapback probe at the 5′end, and the conventional annealing primer at the 3′ end. The sequence of snapback probe is complementary to the target sequence containing mutational sites. After asymmetric PCR, firstly double-strand DNA fragment was generated and then when the limiting primer run out, the excess snapback primer produced single strand amplicon with a snapback probe tail, which led to the formation of the stem-loop hairpin of its own extension product. In the presence of saturating fluorescent DNA dye, the melting peaks of both the stem-loop hairpins and double-strand DNA amplicons can be differentiated by plotting the negative derivative of fluorescence versus the melting temperature (dF/dT). Initially, we designed the snapback primer completely matched to the wild-type allele. If the hairpin stem includes different nucleotide variations, there will be a genotype-dependent melting temperature transition. When rapid PCR protocol with transient denaturation/annealing/extension duration was executed, the product with mismatched allele can be preferentially amplified compared with the product with matched allele.

Seven prepared artificial plasmids were analyzed for KRAS mutations using the Snapback primer system. When the snapback probe assays were optimized, the templates harboring different mutations could be easily discriminated from each other. The melting peaks of a stem-loop hairpin with G12A, G12D, G13D, G12S, G12V, and G12C were observed at 69.25±0.1°C, 67.5±0.2°C, 68.65±0.1°C, 66.5±0.2°C, 66.15±0.3°C, and 67. 75±0.0°C, respectively (Figure 3(b)).

To confirm the assay's discriminating power for single base variation, we artificially synthesized the plasmid fragments with 2 and 3 nucleotide alterations in KRAS codons 12 and 13. Under the same reaction conditions, the melting peaks of the three kinds of plasmid fragment (G13D, GGT>GCA, and GTG>CAC) can be clearly differentiated with the at least 2-degree difference in the melting peaks (Figure 3(c)).

Serial dilutions of KRAS G12A mutant plasmid containing 100%, 10%, 1%, and 0% mutant allele were amplified by snapback PCR and normal PCR primers respectively and then subjected to Sanger sequencing. According to the sequencing signals quantified by the relative fluorescent signal intensity, the abundance of the mutant allele was significantly enriched 10-fold during the snapback primer PCR. As was observed in Figure 3(d), 1% dilution of the KRAS G12A mutant allele can be detected after PCR by snapback primer, while the same sequencing signal of a mutant allele can only be observed in the PCR products with 10% mutant allele load.

### 3.4. Sensitivity of Snapback Primer HRMA for KRAS Mutation Detection

**Figure 4:**
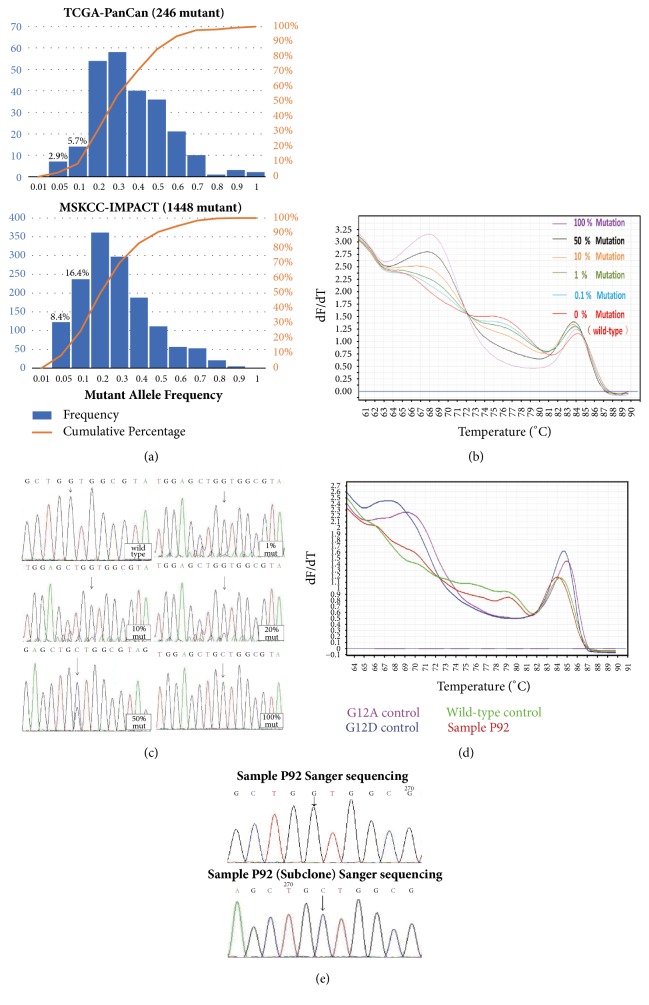
Detection of the KRAS codons 12 and 13 mutations in CRC tissue DNA using snapback primer HRMA. (a) The KRAS mutant allele frequency distribution in TCGA and MSKCC-IMPACT. A number of samples harboring KRAS mutation was dichotomized by mutant allele frequency. The distribution of KRAS mutant samples was skewed in samples with <20% mutant allele. Compared with the primary tumor samples, tissues from metastatic tumors have lower mutant allele burden. (b) Analytical sensitivity of snapback primer HRMA. The system was able to discriminate 1% dilution of KRAS mutant allele from the wild-type sequence. (c) Analytical sensitivity of Sanger sequencing. The direct sequencing can detect KRAS mutation in the plasmid pool with 20% KRAS mutant allele. (d) Patient (P92) with low-abundance G12A mutation was detected using the snapback primer system. (e) Sanger sequencing was determined P92 as KRAS mutation free, while further subclone sequencing of P92 was detected the mutation in the sample.

The sensitivity of the snapback primer HRMA was evaluated by detecting serial dilutions of the KRAD G12D mutant plasmids DNA at a serial dilution of 100%, 50%, 10%, 1%, 0.1%, and 0% (100% wild-type control). Meanwhile, the prepared dilution of the mutant plasmids was also subjected to Sanger sequencing to assess the sensitivity of KRAS mutation detecting by direct sequencing. As was showed in Figures 4(b) and 4(c), up to 1% of the KRAS mutations can be discriminated from the wild-type plasmids after the snapback primer amplification and melting curve analysis. However, the sensitivity of Sanger sequencing is approximately 20% mutation load.

### 3.5. Detecting KRAS Mutations in Colorectal Cancer Tissue Specimens by Snapback Primer HRMA

We screened the KRAS codons 12 and 13 mutations using the DNA samples extracted from 100 CRC tissue specimens using both the snapback primer HRMA and Sanger sequencing. A total of 35 cases were identified KRAS mutation positive as detected by snapback primer HRMA, while 28 cases were found to be KRAS mutation positive by Sanger sequencing (Table 2). Of the 28 KRAS mutation identified by sequencing, there were 21 cases with codon 12 mutation including 12 cases of G12D, 8 cases of G12V, 1 case of G12C, and 7 cases with codon 13 mutation (G13D). The discrepancy between the two methods was verified by T-A cloning sequencing. The representative example of discrepancies was shown in Figures 4(d) and 4(e). All the results of snapback primer HRMA were consistent with those from T-A cloning sequencing.

### 3.6. Precision of the Snapback Primer System for KRAS Mutation Detection

As shown in Table 3, we determined the intra-assay precision as less than 0.3% (CV) and the inter-assay precision as less than 0.5% (CV) by detecting the variance of the mutant allele melting temperature using plasmids with 1% burden of KRAS G12D or G13D mutation,

## 4. Discussion

Over the past decade, cancer treatment has achieved a significant progress towards more personalized targeted therapies. KRAS is the most frequently mutant gene in the RAS/RAF/MAPK pathway, mainly occurring in PAAD, COAD/READ, and LUAD. Recent studies have shown that the patients harboring KRAS mutation were prone to confer resistance to anti-EGFR therapies through the TRK independent hyperactivation of the RAF/MEK/ERK and PI3K/AKT/mTOR pathways signaling. The latest major clinical guidelines have required the KRAS mutation analysis prior to administration of cetuximab and panitumumab [8, 9, 11, 12]. Consequently, KRAS mutations testing is routinely implemented into the clinical practice of CRC patients management.

Large-scale studies of the cancer genome, such as the Pan-Cancer analysis project of The Cancer Genome Atlas have shed light on the route of deciphering cancer genetic codes [28]. However, the mutational status of the KRAS gene across different cancer types still remains largely veiled. In this study, we assessed the prevalence of KRAS genetic alterations, including point mutations, copy number alterations, and gene fusion in both the primary tumors (TCGA) and metastatic tumor samples (MSKCC-IMPACT) at the Pan-Cancer scale. It is not surprising that somatic mutations are the most prevailing type of genetic alterations in the KRAS gene, and codons 12 and 13 are the dominating mutation hotspot across the whole gene. Interestingly, we found the KRAS mutation spots in COAD are more dispersed compared with PAAD and LUAD, which are the other two cancer types with over 20% KRAS frequency. This phenomenon may attribute to the popular mismatch repair deficiency in COAD that cause the microsatellite instability of the cancer genome [29]. Besides the gain-of-function mutations in the gene, KRAS amplification is also found in 1 out of 5 patients with testicular germ cell tumor or ovarian cancer; notwithstanding, the high prevalence of the gene amplification may result from different types of molecular machinery, as the TGCTs have the genetic nature of chromosome 12 amplification [30], while the prevalent TP53 deficiency in OVs can lead to a tolerance of increased gene load [31]. Likewise, somatic mutation is the predominately observed type of genetic alteration in metastatic cancers, and the mutation spots are mostly confined in the codons 12 and 13. On the contrary, the mutational types are more varied in the metastatic cancers, as inframe mutations and gene-fusion are found in the MSKCC-IMPACT cohort.

In this study, we found no difference between the survival of the patients with KRAS mutant primary tumors and those with KRAS wild-type primary tumors in regard to the cancer types of PAAD, COAD, and LUAD, although the prognosis value of KRAS mutation in these cancer types still remains controversial throughout different studies [32–34]. However, KRAS mutant metastatic tumor showed poorer survival versus their KRAS wild-type counterparts (P=4.285 × 10^−6^), further suggesting the importance of KRAS mutation detection in the clinical management of metastatic tumors.

Currently, there are three categories of mainstream approaches for KRAS mutation detection in cancer samples, such as DNA sequencing (Sanger sequencing and high throughput paralleled sequencing), fluorescent probe-based real-time PCR (TaqMan probe, molecular beacon, etc.), and droplet digital PCR (ddPCR). Although sequencing remains the gold standard for mutations detecting by directly getting the genetic sequence, it is both time and labor consuming. The high throughput sequencing technologies are intentionally developed to profile mutations in the multigene panel other than the mutations in a single gene. Sanger sequencing is relatively cost-efficiency friendly for mutation detecting in a focused region of genome; yet this first generation of DNA sequencing technology lacks the sensitivity to detect rare mutations with less than 10% mutant allele burden (Figure 4(c)), which may result in around 10% false negative results in the primary tumors and even over 20% false negatives in the metastatic tumors (Figure 4(a)). Fluorescent probe-based real-time PCR (Therascreen KRAS RGQ PCR kit from Qiagen and Cobas KRAS kit from Roche Diagnostics)[20, 21] and ddPCR (KRAS G12/G13 screening kit from Bio-Rad) are robust tools for identifying rare mutations with down to even 0.01% mutant allele frequency sensitivity[28]. However, since these commercial kits are manufactured with pre-designed probe sequence, they can only detect 7 specific types of common mutation (G12A,G12C,G12D,G12R,G12S,G12V,G13D), which are far less than the 17 mutation types we've identified in the TCGA and MSKCC-IMPACT patient cohort.

The snapback primer assay was initially developed for genotyping the single nucleotide polymorphism (SNP) by the Wittwer Lab in 2008 [15]. Soon afterward, the snapback primer was modified for the rare allele enrichment and detection [24]. The mechanism of genotyping by snapback primer mainly relied on the thermostability of the stem-loop hairpin, which can be determined by the hairpin loop size, stem length and most importantly, the base mismatches within the stem. Since different nucleotide variations would produce unique melting curve profile during melting curve analysis, the snapback primer mediated HRMA can theoretically identify almost all mutations throughout the probe region.

In this research, we developed a robust snapback primer assay to detect mutations within the codon 12 and 13 of KRAS gene. Although some similar multiplex assays have been reported to detect these mutations [35, 36], there are still major technical defects such as the demand for further genotyping via sequencing analysis or multiplex labeled probes, resulting in an increased risk of DNA contamination or reduced robustness of the assay. In our snapback primer HRMA system, we used one pair of snapback-limiting primer set without any special covalent modifications to enrich and detect all potential mutations in the codons 12 and 13 of KRAS gene in a one-tube PCR system. After 10-fold mutation allele enrichment during snapback primer guided rapid PCR, the sensitivity of the assay was around 1% mutant allele load, which is below the minimum mutation load (0.05) in both the TCGA and the MSKCC-IMPACT database (Figure 4(a)). All this evidence suggested that our method is a promising method for the mutation screening in codon 12 and 13 compared with the conventional methods such as Sanger sequencing, which may cause 10-20% false negatives in the final results.

The accuracy of the snapback primer assay was evaluated with DNA samples extracted from 100 CRC tissue specimens in comparison with directing sequencing. A total of 7 discordant results were observed between these two methods, which are all KRAS mutation positive in snapback primer HRMA but mutation negative in Sanger sequencing. As validated by the further T-A cloning sequencing, all these 7 discordances were yielded by false negative from Sanger sequencing.

However, our present study had several limitations. Firstly, the survival analysis needs to be more deliberately stratified with the clinical indexes such as disease staging, drug, and radiological treatment records, so as to maximize the clinical relevance of the KRAS mutations. Secondly, our KRAS mutation analysis used the tumor genetic codes obtained from NGS sequencing, with an average sequencing depth around 100×, which may yield false negative mutation callings or mosaiced mutational allele estimation in cancer types with extremely low tumor cellularity. Thirdly, since the snapback system enriches the mutant allele in the sample before the downstream HRMA detection of the mutations, it compromises the relative quantification of the mutation load which can only be assessed by the relative height of mutation melting peak versus wild-type melting peak. Fourthly, the reproducibility of this assay needs further validation on different fluorescent PCR platforms. The snapback primer system was mainly optimized on RoterGene Q platform, yet its application on other platforms was uncertain. In addition, although the assay for the detection of KRAS mutation was characterized as 1% dilution of the mutant allele, the actual limit of detection in different types of clinical specimens still requires further study.

## 5. Conclusion

KRAS mutation is a founder mutation in cancer. Although somatic mutation in codons 12 and 13 is the dominating kind of KRAS genetic alteration, the genetic background of KRAS mutations harbors a high diversity across different cancer types, especially in the metastatic cancer tissues. Moreover, KRAS mutation is strongly associated with the poor survival of metastatic cancers. Mutant allele frequency analysis revealed the skewed distribution of KRAS mutant samples with less than 20% mutant allele. Snapback primer HRMA is a reliable, highly sensitive, high-throughput for detecting the common KRAS mutations in tumor samples obtained from cancer patients.

## Figures and Tables

**Table 1:** Summary of samples with simultaneous KRAS mutation in TCGA and MSK-IMPACT cohort.

**Cancer Study**	**Sample ID**	**Tumor Tissue**	**AA Change**	**Start Pos**	**End Pos**	**Reference**	**Variation**	**Mutant Allele Freq**
TCGA	TCGA-2J-AAB1-01	Pancreas	G12D	Chr12:25398284	Chr12:25398284	C	T	NA
			G12R	Chr12:25398285	Chr12:25398285	C	G	0.13
TCGA	TCGA-2J-AABH-01	Pancreas	G12V	Chr12:25398284	Chr12:25398284	C	A	0.19
			G12S	Chr12:25398285	Chr12:25398285	C	T	0.19
TCGA	TCGA-FB-A78T-01	Pancreas	G12A	Chr12:25398284	Chr12:25398284	C	G	0.14
			G13C	Chr12:25398282	Chr12:25398282	C	A	0.15
TCGA	TCGA-YB-A89D-01	Pancreas	G12D	Chr12:25398284	Chr12:25398284	C	T	NA
			G12R	Chr12:25398285	Chr12:25398285	C	G	0.17
MSK-IMPACT	P-0002475-T01-IM3	Metastatic Tumor	G12D	Chr12:25398284	Chr12:25398284	C	T	0.31
			G13D	Chr12:25398281	Chr12:25398281	C	T	0.38
MSK-IMPACT	P-0006073-T01-IM5	Metastatic Tumor	G12D	Chr12:25398284	Chr12:25398284	C	T	0.05
			G12C	Chr12:25398285	Chr12:25398285	C	A	0.13
MSK-IMPACT	P-0006634-T01-IM5	Metastatic Tumor	G13D	Chr12:25398281	Chr12:25398281	C	T	0.02
			G12I	Chr12:25398284	Chr12:25398285	CC	AT	0.15
MSK-IMPACT	P-0008464-T02-IM5	Metastatic Tumor	G12V	Chr12:25398284	Chr12:25398284	C	A	0.03
			G12R	Chr12:25398285	Chr12:25398285	C	G	0.20
MSK-IMPACT	P-0009837-T01-IM5	Metastatic Tumor	G12F	Chr12:25398284	Chr12:25398285	CC	AA	0.05
			G12C	Chr12:25398285	Chr12:25398285	C	A	0.13
MSK-IMPACT	P-0010007-T01-IM5	Metastatic Tumor	G12V	Chr12:25398284	Chr12:25398284	C	A	0.05
			G12A	Chr12:25398284	Chr12:25398284	C	G	0.17
MSK-IMPACT	P-0011012-T01-IM5	Metastatic Tumor	G12C	Chr12:25398285	Chr12:25398285	C	A	0.03
			G12S	Chr12:25398285	Chr12:25398285	C	T	0.06

**Table 2:** Distribution of KRAS mutations detected by Snapback Primer-based HRM, Sanger sequencing, and T-A Cloning Sequencing in 100 CRC tissues.

Methods	G12D	G12V	G12C	G12A	G13D	Total
Snapback primer-based HRM	14	10	1	1	9	35
Direct sequencing	12	8	1	0	7	28
T-A Cloning Sequencing	2	2	0	1	2	7

**Table 3:** Intra- and interassay precision of the snapback primer system for KRAS mutation detection.

	Intra-assay		Inter-assay	
	G12D	G13D	G12D	G13D
Mean (Melting temperature)	67.6	68.5	67.5	68.7
Standard Deviation	0.16	0.15	0.29	0.31
Coefficient of variance (%)	0.23	0.22	0.43	0.45
